# Adaptation, diversification and ecological opportunity in the extremophile radiation of *Zoarcoidea*

**DOI:** 10.1098/rspb.2025.1289

**Published:** 2025-08-27

**Authors:** Christine Thacker

**Affiliations:** ^1^ Vertebrate Zoology, Santa Barbara Museum of Natural History, Santa Barbara, CA, USA; ^2^ Ichthyology, Natural History Museum of Los Angeles County, Los Angeles, CA, USA

The polar seas and deep oceans are characterized by extremes in temperature, sunlight and pressure, yet they are inhabited by some of the most rapidly diversifying fishes on earth [1–3]. These fishes can survive in challenging environments due to a suite of adaptations, most notably the presence of antifreeze proteins or glycoproteins (AFP/AFGP) that insulate the blood from ice formation at subzero temperatures [4–6]. Perciform fish clades, including the Antarctic icefishes, plunderfishes and dragonfishes (*Cryonotothenioidea*), the snailfishes (*Liparidae*), and the eelpouts, pricklebacks, gunnels and wolffishes (*Zoarcoidea*), comprise the bulk of the fishes in polar oceans. All these groups express AFPs or AFGPs in their blood and have undergone accelerated species diversification compared to their close relatives, characteristics of adaptive radiation [3,7,8]. A new study by Brownstein *et al.* [7] is a detailed examination of the lineage evolution in *Zoarcoidea*, unravelling a radiation that features a complex interplay of key traits, ecological opportunity, and species diversification.

## Theoretical framework of adaptive radiation

Adaptive radiation is defined as an evolutionary innovation in a monophyletic group (clade) which precedes and facilitates rapid species diversification that is usually linked to invasion of a novel habitat (ecological opportunity) [9,10]. Adaptive radiations may also be characterized by an early burst pattern of change in both phenotypic disparification and lineage diversification, in which proliferation occurs rapidly and then levels off, potentially due to saturation of the novel niche [9,10]. Known examples of adaptive radiation are often more complex; they may proceed in stages, they may involve multiple trait innovations acting synergistically, and there may be a lag time between the origin of novel traits and an increase in diversification [10]. The availability of ecological opportunity is also a crucial factor in promoting species radiation [9]. As phylogenomic investigations of species radiations accumulate, it is also becoming clear that genomic variation is common and often present in clades that feature accelerated diversification. Genomic variation can include gene duplication, variation in substitution rate and gene copy number and increases in transposable elements, all of which have been linked to increased species diversification [5,6,11]. Specific causes for accelerated diversification may only be inferred in hindsight, and so the standing genomic variation that does not lead to an adaptive radiation, or has not yet led to one, may be overlooked. The temporal patterns of phenotypic and genomic changes, ecological transitions and diversification accelerations can be established based on a calibrated phylogenetic tree but the causal links among those events are difficult to infer, particularly in complex radiations involving multiple traits, invasions, and species radiations.

## Adaptive radiations of fishes at the poles

The classic example of a polar adaptive radiation is *Cryonotothenioidea*, a single clade of southern hemisphere fishes that possesses AFGPs and has invaded and radiated within the Southern Ocean around Antarctica. Brownstein *et al*. [7] examine a more complex evolutionary pattern among lineages of *Zoarcoidea*, a group of elongate, benthic fishes, predominantly predators, that are most abundant in cold temperate boreal seas. Zoarcoids have also invaded both poles and the deep sea multiple times. Brownstein *et al*. [7] use genomic ultraconserved element sequences and several novel zoarcoid fossils to infer phylogeny and construct a detailed timescale of trait evolution, species diversification and habitat transitions. They then examine how these events concur with paleoclimatic patterns that have had particular impact on polar habitats and the deep sea.

In *Zoarcoidea*, the innovation of AFPs occurred in the ancestor of all living taxa (minimum crown age estimate of 25 Ma) and was followed by an increase in diversification at roughly 18 Ma that is seen in the phylogeny as a series of short internodes among clades (the ‘anomaly zone’, where a subset of gene trees strongly supports an alternate topology) and is also identified as a rate shift by both the BAMM and TESS CoMET methods [3,7]. An additional nested diversification acceleration within the family *Zoarcidae* occurred at around 6.2 Ma, detected by BAMM and TESS CoMET in a broader analysis of acanthomorph fish diversification [3], and this rate shift is concordant with another anomaly zone in the hypothesis of Brownstein *et al*. [7]. *Zoarcidae* is the most diverse zoarcoid family and the only one that includes invasions of both poles; several lineages within *Zoarcoidea* have dispersed into Arctic seas but only *Zoarcidae* is known from the Antarctic [12]. Invasions of polar habitats and the deep sea occurred between 5 and 8 Ma and phylogenetic optimization of dispersal patterns indicates that two strong peaks of founder event colonization occurred over the past 10 Ma. At around the same time (5–10 Ma), zoarcoids show a pulse of phenotypic trait changes, including traits related to feeding (burrowing as camouflage for ambush predation, tooth morphology) and reproduction (ovoviviparity), as well as the presence of conjoined dorsal, caudal and anal fins, facultative air breathing and association of juvenile fishes with scyphozoan jellies. This pulse of trait diversification is found throughout *Zoarcoidea* and is not exclusive to the extremophile lineages, and the traits involved do not have any clear linkages to survival in extreme habitats. However, the presence of a recent pulse of novel traits among zoarcoids does indicate that the group continues to actively diversify [7].

## Time lags between adaptation, ecological opportunity and species radiation

The sequence of events throughout zoarcoid evolution is consistent in some ways with adaptive radiation but is unique in that the group contains two nested radiations with an attenuated timescale between the origin of AFPs, diversification accelerations, periods of global cooling and transitions into polar habitats and the deep sea. Significant cooling of the earth’s polar regions and the onset of Antarctic glaciation began around 33 Ma at the Eocene–Oligocene transition [4,7], and AFPs arose in *Zoarcoidea* between that time and the origin of the extant crown taxa at 25 Ma. At roughly 14 Ma, the Middle Miocene Climate Transition (MMCT) ushered in another period of global cooling and expansion of ice sheets at both poles that continued throughout the late Miocene and Pliocene and was particularly intense during the Pleistocene glaciations [4,7,12]. The polar and deep sea invasions among *Zoarcoidea* occurred between 5 and 8 Ma, concurrent with that period of planetary cold conditions. Curiously, there is a lag of at least 7 million years separating the origin of AFPs in *Zoarcoidea* and their first pulse of accelerated diversification in boreal seas, followed by an additional 11 million years before the second diversification uptick and the invasions of polar and deep sea habitats. Zoarcoids also deviate from the classic adaptive radiation framework in that they do not show an early burst of either species or morphological diversification. Acceleration in species diversity shows a steady increase from the Miocene to the present and transitions into novel regions of shape morphospace are phylogenetically assorted, without evidence of a jump of within-clade disparity concordant with the onset of elevated diversification [7]. The polar radiations within *Zoarcoidea* may still be underway.

In both *Zoarcoidea* and *Cryonotothenioidea*, the origin of antifreeze capability is separated from the advent of accelerated species diversification and the later invasions of polar habitats by roughly 7−18 million years [5–7]. The time lags between the origin of a relevant adaptation, the opportunity for habitat invasion, and the onset of increased diversification in polar groups are comparable to those seen in fishes invading freshwater habitats on oceanic islands [13]. A phylogenomic analysis of all acanthopterygian fishes identified 27 instances of accelerated diversification in groups as varied as butterflyfishes, rockfishes, flatfishes, cichlids, wrasses and gobies, along with the polar clades of *Zoarcoidea* and *Cryonotothenioidea* [3]. All those radiations may be linked to invasion of novel habitats, but identification of precipitating traits is often less clear. Rockfishes (*Sebastes*) of the Eastern Pacific are the most rapidly radiating acanthopterygian lineage and potential key traits for this radiation are fine habitat segregation by depth and livebearing. These factors may plausibly be linked to isolation of populations and promotion of speciation, and they coincide evolutionarily with the increase in species diversification [3]. However, most of the radiations display temporal gaps between the origin of a key trait and the advent of accelerated diversification. Among flatfishes, the key innovation of eye migration is not immediately associated with radiation; the advent of asymmetric eye placement is separated from accelerated diversification by a gap of at least 12 million years [3]. In radiations such as darters (*Ethostomatinae*), guppies (*Poeciliidae*) and grunters (*Terapontidae*), novel parental care strategies are present, but it is likely that most of their diversification is due to invasion of highly fragmented freshwater habitats.

A variety of ecological, reproductive, and metabolic innovations may plausibly be invoked as causes for pronounced species diversification among fishes, and in many cases the innovations are separated from novel ecological opportunities and diversification radiations by gaps of millions of years. Temporal gaps may be due to multiple factors including the accumulation of sequential trait innovations, delay in habitat availability due to climatic or tectonic changes and invisible but vital genomic change [10,11,13,14]. In *Cryonotothenoidea*, additional adaptations beyond the evolution of AFGPs precede their invasion of the Southern Ocean, including the loss of heat shock gene expression, reduction in skeletal ossification and increases in lipid deposits [5,14]. Cryonotothenioid species lack swim bladders, and the skeletal reductions and lipid accumulation serve to enhance buoyancy and facilitate invasions of the water column from an ancestrally benthic habitat [14].

## Significance of genomic variation and adaptation

Structural genomic changes are also associated with polar adaptive radiations. Signatures of antifreeze gene copy expansions and contractions in different thermal environments have been identified in both *Zoarcoidea* and *Cryonotothenioidea*, with lineages in colder habitats exhibiting more extensive duplications than those in warmer waters [5,6]. AFP gene complements in *Zoarcoidea* have undergone repeated expansions and contractions as lineages transitioned between polar and cold temperate habitats, with many instances of gene duplication and translocation evident in whole genomes [6]. In *Cryonotothenioidea*, the time prior to Antarctic invasion is characterized by a high rate of overall molecular change, diversification of genes associated with reduced bone density, and extensive expansions of antifreeze genes [5,14]. The evolutionary patterns and tempo of polar invasions in *Liparidae* are not known, but the genome of a hadal *Pseudoliparis* species is expanded relative to snailfishes in shallow habitats, with duplications in metabolic and osmoregulatory genes that facilitate survival in the deep sea [15].

The detailed evolutionary analysis provided by Brownstein *et al*. [7] shows that the nested radiations of *Zoarcoidea* have probably resulted from a complex interplay of adaptations, genomic modulations, climate changes and opportunities for habitat invasion, in which any one of those factors is necessary but not sufficient to yield an increase in species diversification. Their work joins a growing body of empirical studies that are revealing the genomic bases of adaptive radiation and underscoring the importance of transitions to novel habitats and ecological opportunity as drivers of bursts of species diversification [4,7,12,13]. Even more compelling, evidence is accumulating that lags between the acquisition of trait innovations and diversification pulses are common, with novel genotypic and phenotypic traits quietly accumulating and setting the stage for radiation but not triggering it. It may be that standing genomic variation is sufficient to guarantee that candidates for adaptive radiation are abundant, each one poised to exploit novel and unpredictable ecological opportunities when they arise. A saying attributed to the Roman philosopher Seneca asserts that ‘luck is what happens when preparation meets opportunity’. In the polar adaptive radiations of *Zoarcoidea* and *Cryonotothenioidea*, the luck of species diversification results when the preparation of advantageous adaptations and genomic variation meets the opportunity of novel habitats and environmental change.

## Data Availability

This article has no additional data.
